# Femtosecond Laser Fabrication of Wettability-Functional Surfaces: A Review of Materials, Structures, Processing, and Applications

**DOI:** 10.3390/nano15080573

**Published:** 2025-04-09

**Authors:** Zelin Chen, Jiantao Zhou, Wenyang Cen, Yinzhou Yan, Wei Guo

**Affiliations:** 1 Ningbo Institute of Materials Technology and Engineering, Chinese Academy of Sciences, Ningbo 315201, China; chenzelin@nimte.ac.cn (Z.C.); zhoujiantao@nimte.ac.cn (J.Z.); cenwenyang@nimte.ac.cn (W.C.); 2 School of Physics and Optoelectronic Engineering, Beijing University of Technology, Beijing 100124, China; yyan@bjut.edu.cn

**Keywords:** femtosecond laser, micro/nano structures, functional surface, wettability

## Abstract

Wettability-functional surfaces are crucial in both theoretical investigation and engineering applications. Compared to traditional micro/nano fabrication methods (such as ion etching, sol–gel, chemical vapor deposition, template techniques, and self-assembly), femtosecond laser processing has unique advantages, such as unmatched precision, flexible controllability, and material adaptability, widely used for the fabrication of wettability-functional surfaces. This paper systematically discusses the principle and advancement of femtosecond laser micro/nano processing in regulating surface wettability and analyzes the laser modulation mechanisms and structural design strategies for wettability-functional surfaces on various materials. Additionally, this paper reviews the practical applications of femtosecond laser-based wettability-functional surfaces in environmental engineering, aerospace, and biomedical fields, while highlighting the challenges and future directions for femtosecond laser processing of wettability-functional surfaces.

## 1. Introduction

**Figure 1:**
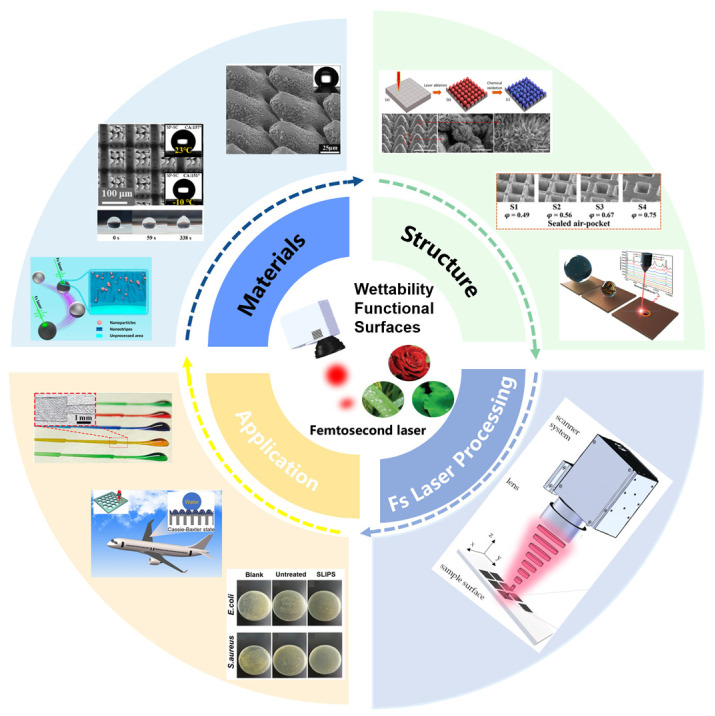
Design and application of wettability-functional surfaces via femtosecond laser processing.

Wettability serves as a key material property that defines the interaction between a solid surface and a liquid medium, primarily determined by the stability of the gas–liquid–solid phase interface [1,2,3,4,5]. Recently, the cognition and further regulation of surface wettability has attracted much attention attributed to the development of bio-inspired technology, closely related to the intrinsic material properties, multi-scale structures, efficient fabrication, and industrialized applications [6,7,8]. Through micro/nano structures, the surface wettability can be smartly controlled based on functional surfaces [9]. Functional structures are currently fabricated using top-down or bottom-up methods, such as stencil [10], etching (wet etching or dry etching) [11], and laser direct writing [12]. Among these methods, laser processing is a contact-free method that can easily focus on micro/nano scales and avoid complex masks [13]. However, the laser-based fabrication of micro/nano structures is still suffering bottlenecking for the widespread adoption of wettability-regulated surfaces due to the limitation of resolution related to the wavelength [14]. Compared with long pulse laser, femtosecond laser has unique advantages in spatial processing with high precision owing to the ultrahigh peak power (10^12^ to 10^15^ W/cm^2^) and ultrashort pulse (10^−15^ s), effectively avoiding obvious thermal damage, suppressing the heat effects of laser ablation, and enabling “cooling” processing to a certain extent [15,16,17,18]. Based on the ablation effect, the surfaces can be further modified by femtosecond direct writing with high flexibility when the laser energy exceeds the threshold value. Moreover, femtosecond lasers can be applied to nearly all materials, enabling precise micro/nano structure fabrication on surfaces. However, the femtosecond laser processing of functional surfaces still suffers from several problems, such as considering the stimulation of external fields and the fabrication ability and efficiency of extremely small structures beyond the light optical diffraction limits [19,20]. In past decades, spatial light modulation has attracted much attention for the balanced quality and efficiency of femtosecond laser-processed functional surfaces [21]. This review introduces the basic principles and special wettability models and elaborates on the research progress of femtosecond laser processing of different material types (such as intrinsic hydrophilic/hydrophobic materials, smart wettability-switching materials) and different micro/nano structure types (such as uniform and non-uniform structures). Additionally, this paper highlights the current modulation of femtosecond laser and further industry applications of fabricated wettability-regulated surfaces in the environmental engineering, aerospace, and biomedicine fields. This review follows the frame of “material-structure-processing-application” for femtosecond laser-based wettability regulation and provides insight into the outlook of further development. Figure 1 summarizes the progress of femtosecond laser fabrication of wettability-functional surfaces on various materials and the creation of different structural types for these surfaces, highlighting their applications in various fields.

## 2. Theoretical Basis of Surface Wettability

**Figure 2:**
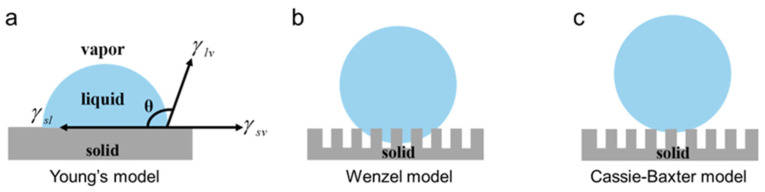
Basic concepts related to surface wettability and several typical wettability models. (**a**) Young’s model for flat substrates [24]; (**b**) Wenzel model for rough substrates [25]; (**c**) Cassie–Baxter model avoiding penetration [26].

As well known, wettability reflects the ability of liquid to spread and diffuse on solid surfaces. The balanced energy between the solid–liquid interface promotes the spreading of the liquid. Meanwhile, the surface tension significantly resists the spreading process. The contact angle (CA) is typically employed for quantifying the wettability (Figure 2). The surface is considered superhydrophilic when the contact angle is below 10° and superhydrophobic when it exceeds 150° [22,23].

(1) Young’s Model [24]: The relationship between the contact angle (*θ*) and the surface tension of the liquid–gas interface, solid–gas interface, and solid–liquid interface, known as Young’s equation, can be numerically expressed as:

(1) $$\gamma_{\mathrm{sl}}=\gamma_{\mathrm{sg}}-\gamma_{\mathrm{lg}}\cdot \cos\theta$$

where *γ*_lg_, *γ*_sg_, and *γ*_sl_ denote the surface tension of the liquid–gas interface, solid–gas interface, and solid–liquid interface, respectively.

(2) Wenzel Model [25]: For non-ideal surfaces with uneven chemical composition and morphology, Young’s equation cannot be utilized to describe wetting behavior. Thus, Wenzel et al. further modified Young’s equation and proposed that droplets penetrated the rough structure on the surface. In the Wenzel state, the contact angle θ* is given by the Wenzel wetting equation, which largely depends on the contact angle *θ* from Young’s equation.

(2) $$\cos\theta^{*}=r\cos\theta$$

where *r* represents the ratio of the effective solid surface area and the projected area of rough surface. The Wenzel wetting equation is particularly suitable for describing hydrophilic surfaces, revealing that the contact angle θ* can be reduced by increasing surface roughness. However, this model is unable to describe hydrophobicity because of a lack of ability to account for the air layer between the droplet and surface.

(3) Cassie–Baxter Model [26]: Cassie and Baxter introduced another model to explain the wetting phenomena, such as the lotus effect and self-cleaning behavior. This model assumes that in hydrophobic conditions, the droplet does not penetrate the surface roughness but instead forms a three-phase composite contact between the solid–liquid and gas–liquid interfaces. As a result, a new contact angle relationship is introduced as:

(3) $$\cos\theta^{*}=f_{1}\cos\theta_{1}+f_{2}\cos\theta_{2}$$

where *θ*_1_ and *θ*_2_ are the Young’s contact angles of the droplet on the solid and gas phases, respectively. *f*_1_ and *f*_2_ are the percentages of the solid–liquid and gas–liquid contact areas (*f*_1_ + *f*_2_ = 1). Therefore, when *θ*_2_ = 180°, Equation (3) is:

(4) $$\cos\theta^{*}=f_{1}\cos\theta_{1}+f_{1}-1$$

In the Cassie state, the air layer between the droplet and the material surface significantly reduces the contact area between the liquid and the solid surface, making the three-phase contact line of the droplet discontinuous. This condition is more favorable for the droplet to roll on the solid surface.

(4) Other models considering mediums [27]: In addition to three typical wettability models mentioned above, various wettability states have also attracted significant attention. Due to the different interactions between mediums and surfaces, factors such as liquid surface tension and intermolecular forces can affect the affinity of the surfaces. Therefore, consideration of the composition of the mediums is necessary. For example, ordinary hydrophilic, hydrophobic, oleophilic, and oleophobic states will be introduced in the air. The gas-related wettability states, such as superaerophilicity and superaerophobicity, also emerge in the water. Generally, these states are obtained by combining the intrinsic wettability of flat substrates (hydrophilicity, hydrophobicity, oleophilicity, and oleophobicity) with super-wettability after introducing micro- and nanoscale roughness in different mediums (air, water, and oil), inducing a total of 64 unique wettability states and providing a rich foundation for the application of super-wettability surfaces. The transition between different wettability states can be achieved through a combination of micro/nano structure design, surface chemical modification, composite structures with liquid injection, and the integration of stimulus-responsive materials and structures. These wettability states can offer abundant possibilities for the design of surface wettability.

## 3. Materials with Determinative and Switching Wettability

According to the above-mentioned Young’s equation and related theories, the wettability is affected by the chemical properties and surface micro/nano structures. For materials with different intrinsic wettability, such as hydrophilic materials with high surface energy (e.g., most metals) and hydrophobic materials with low surface energy (e.g., polymers), surface wettability design should be subsequently tailored.

### 3.1. Materials with Determinative Wettability

#### 3.1.1. Materials with Intrinsic Hydrophilicity

**Figure 3:**
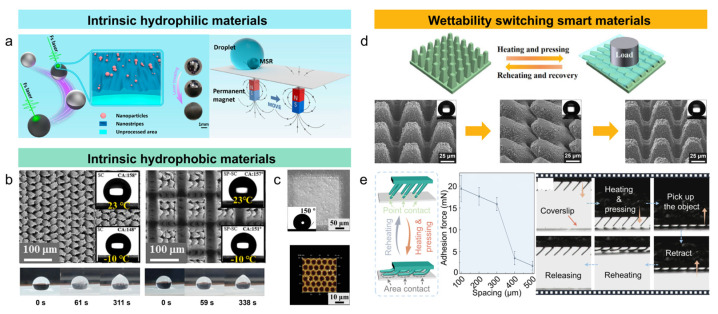
(**a**) Superhydrophilic stainless steel spheres with enabled droplet manipulation and self-cleaning at a magnetic field [29]; (**b**) square pillar and Siberian-cocklebur-like structures on PTFE, related surface morphology, contact angle at different temperatures, and ice delay time [37]; (**c**) micro-pit structures on PDMS with a contact angle of 150° [38]; (**d**) femtosecond laser-induced controllable changes in the surface morphology and wettability of shape memory polymers [49]; (**e**) microcone array state changes enable coverslip capture, transfer, and release [50].

Due to the excellent electrical conductivity, high density, and high mechanical strength, metals are widely used in the aerospace and biomedical fields. Metal surfaces typically exhibit high surface energy, so untreated metal surfaces generally are hydrophilic. Tahir et al. [28] prepared ordered structures with different surface morphologies and sizes on the TiNbZrSn alloy. The processed samples showed a contact angle of 0°, exhibiting superhydrophilicity. He et al. [29] employed femtosecond laser processing to create uniform micro/nano structures on the surface of stainless steel. Experimental results showed that as the laser scanning speed decreased, the oxygen content on the stainless steel significantly increased with increased roughness and decreased water contact angle, demonstrating excellent hydrophilicity (Figure 3a). However, superhydrophilic surfaces are prone to transforming into hydrophobic or superhydrophobic when exposed to the atmosphere due to organic adsorption. Long et al. [30] discovered that the type of functional groups and the carbon chain length significantly affected wettability transition. Medium- and long-chain organic compounds increase the contact angle, promoting the transition from hydrophilic to hydrophobic. Meanwhile, the short-chain organic compounds have a weaker effect and are more easily dissolved in water. The conventional structures are difficult to maintain for long-lasting superhydrophilicity. To address this issue, the micro/nano composite structures can delay the diffusion of organic substances and provide liquid-wetting channels, ensuring superhydrophilicity when contaminated. Laser processing of micro/nano structures on metal surfaces and further modification with low surface energy organic compounds (such as fluoroalkyl silane, chlorosilane, thiol, etc.) can yield surfaces with excellent superhydrophobic properties [31,32,33]. The synergistic design of a micro/nano structure and a low surface energy organic binary compound has been widely applied to the treatment of metal surfaces with specified wettability properties. However, such a design is susceptible to erosion in corrosive environments (e.g., seawater), leading to coating degradation and peeling and significantly reducing the durability of superwettability [34,35]. Yan et al. [36] proposed the technique combining femtosecond laser element doping with cyclic low-temperature annealing and successfully fabricated bionic ant-nest-like structures on an aluminum surface, presenting a complex geometric morphology primarily composed of sub-crystalline phases. The increased surface roughness, enhanced air capture and storage ability, and significantly reduced surface free energy resulted in unique superhydrophobic chemical stability. In a corrosive 3.5 wt.% NaCl aqueous solution, the superhydrophobicity remained stable for about 2000 h with a stabilized contact angle of approximately 158°. The superhydrophobic metal surface was prepared without the need for organic coatings, offering stable gas storage, low surface energy, and high chemical stability and breaking away from the traditional superhydrophobic design relying on organic modifications.

#### 3.1.2. Materials with Intrinsic Hydrophobicity

Polymers, including polytetrafluoroethylene (PTFE) and polydimethylsiloxane (PDMS), are valued for their exceptional flexibility, thermal stability, high optical transparency, non-toxicity, and excellent biocompatibility. Owing to naturally low surface energy, the polymers exhibit intrinsic hydrophobicity. Through femtosecond laser micro/nano structuring, hydrophobicity can be further enhanced, enabling the fabrication of superhydrophobic surfaces. Ge et al. [37] employed a femtosecond laser to create square pillar and Siberian-cocklebur-like structures on PTFE. The microstructures maintained the contact angle of 151° at −10 °C, effectively reducing frost formation and adhesion and preventing water droplets (Figure 3b). Additionally, the ice-repellent performances demonstrated that the water droplets froze much slower on the structured surfaces, and the freezing time extended to 311 s, largely ascribed to the reduced contact area and air gaps within the microstructures, which slowed heat transfer and delayed freezing. Yang et al. [38] used a femtosecond laser with different parameters to fabricate superhydrophobic surfaces on PDMS, achieving contact angles ranging from 110° to 150°. The variation in contact angle was attributed to micro-pit structures with different periodicities, which trapped a large amount of air and reduced the contact area between water droplets and the surface, thus enhancing the water-repellent capability and significantly improving surface hydrophobicity (Figure 3c).

In recent years, biomedical implant materials have shifted from metals to bio-ceramics to address issues like poor chemical stability and biotoxicity. Zirconia ceramics, with their high strength, excellent biocompatibility, and esthetic properties, have emerged as a mainstream choice, widely applied in dental restorations. To enhance surface performance, Zheng et al. [39] utilized femtosecond laser ablation to create a mechanically stable, durable, and self-cleaning superhydrophobic surface on zirconia substrates. Jing et al. [40] employed a nanosecond laser to construct micro/nano structures on zirconia, followed by silane treatment, achieving a wear-resistant superhydrophobic surface. Glass fiber-reinforced polymers (GFRPs), characterized by light weight and high corrosion resistance, have been widely used in the construction, transportation, and aerospace industries. Zhu et al. [41] proposed laser-processed superhydrophobic surfaces and developed grid-patterned micro-hummock structures on GFRP, achieving a 163.9° superhydrophobic surface with delayed ice nucleation and reduced ice adhesion for wind turbine blade applications. Wang et al. [42] also introduced a femtosecond laser-based multi-pulse grid pitting erosion (MP-GPE) technique to fabricate micro/nano crater arrays on GFRP and attained a 160.6° superhydrophobic surface, corroborating theoretical predictions and expanding the precision in hydrophobic surface engineering.

### 3.2. Smart Materials with Switching Wettability

In recent years, strain regulation through the stretching and releasing of soft materials has been widely applied to the fabrication of smart wettability surfaces. For example, Zhang et al. [43] fabricated micro-groove arrays and combined them with uniaxial stretching to regulate the thickness of liquid films based on femtosecond laser etching, enabling reversible adjustment of droplet sliding behavior. Wang et al. [44] controlled the width of micro-grooves on wearable PDMS substrates through finger bending and achieved the reversible switching of droplet wettability from the “lotus” to “rose”. However, the method of switching wettability by externally stretching or bending elastic polymers is difficult to be locally controlled. Moreover, the deformed structure spontaneously recovers when removing the external force, making it challenging to maintain the smart response [45]. Shape memory polymers (SMP), as novel smart materials, can achieve reversible surface morphology transformation from permanent state to a temporary state under external environmental stimuli. The materials have garnered significant attention in the field of smart wettability response [46,47,48]. Bai et al. [49] induced graded micropillar arrays on SMP surfaces using a femtosecond laser, successfully creating superhydrophobic memory surfaces capable of altering the morphology and wettability. When the synthetic surface was subjected to an external load, the deformation of the micro/nano structures weakened the superhydrophobicity because of the tilted micropillars. However, due to the excellent macro/micro shape memory effect of SMP, simple heating can restore the original surface morphology and wettability. Even after 10 cycles of compression heating, the surface can regain the original superhydrophobicity. In addition to recoverability, the laser-induced microstructures on SMP also demonstrated remarkable durability, with the contact angle remaining above 150° (Figure 3d). Li et al. [50] employed a femtosecond laser slanted micro-machining method to create shape memory micro-cone arrays with a tilt angle of 45°. Under external heating or pressure stimuli, the surface structure could transform into a larger bending angle (>45°, collapsed state) and quickly return to the original state when the temperature exceeded the SMP glass transition temperature (Figure 3e). Moreover, the impact of femtosecond laser processing on the microcone spacing, height, and bending angle was further investigated. The wettability and adhesion could be reversed during the switching between the collapsed and tilted states, with the contact angle decreasing from 156° to 133°. Furthermore, to achieve non-contact, remote, and precise control of wettability on SMP surfaces, Wang et al. [51] incorporated photothermal-responsive nanomaterials into shape memory polymers and used laser processing to create superhydrophobic microstructure arrays, resulting in smart wettability with near-infrared (NIR) light responsiveness. Due to the excellent shape memory performance and rapid photothermal response, the micropillar arrays on the surface could reversibly switch between an upright and tilted state under NIR light, thereby regulating the surface wettability. In the upright state, the micro-pillar arrays exhibited low-adhesion superhydrophobicity. In the tilted state, high-adhesion characteristics were exhibited. Cyclic experiments demonstrated that the wettability transition could be repeated multiple times, enabling the capture and remote release of droplets. By selectively irradiating the corresponding region beneath the droplet, different droplets on the same surface could be released. Table 1 summarizes the femtosecond laser processing parameters to create superhydrophilic/superhydrophobic micro/nano structures on various materials.

## 4. Functional Structures for Wettability Control

### 4.1. Uniform Micro/Nano Structures

#### 4.1.1. Single-Scale Structures

**Figure 4:**
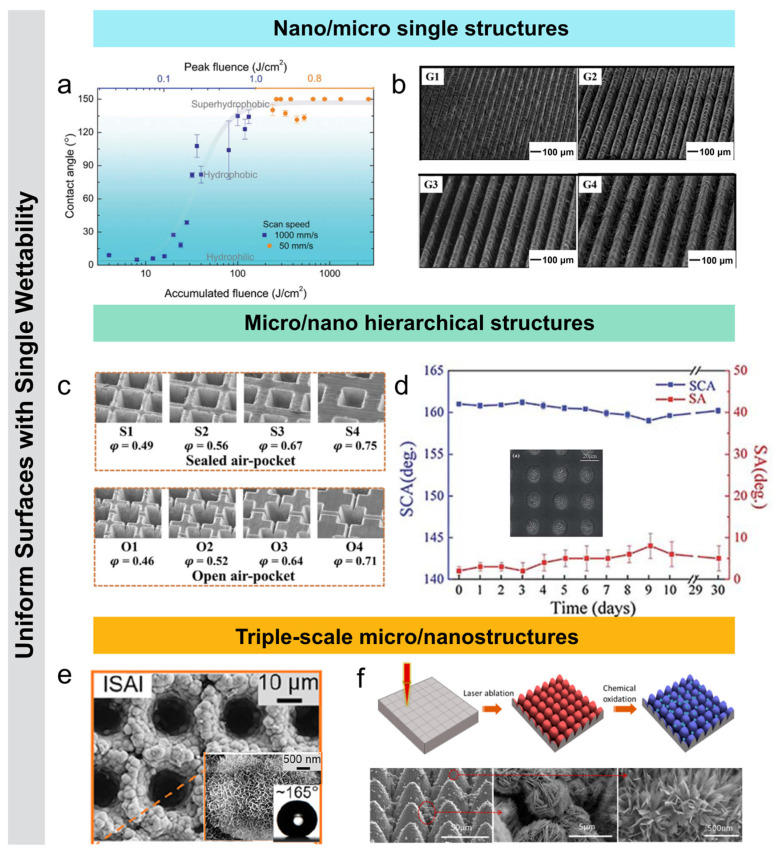
(**a**) Variation in contact angle on stainless steel LIPSS surface with accumulated energy density [68]; (**b**) grooved structures with different periods on aluminum alloy surface by adjusting femtosecond laser spacing [53]; (**c**) air pockets (sealed and open) on Ni surfaces [72]; (**d**) periodic micro-pit arrays and self-organized complex nanostructures on quartz glass surface and the wettability [55]; (**e**) micro-pit arrays, non-uniform micro-clusters, and irregular nanosheet composite structures on aluminum surfaces with excellent superhydrophobic properties [73]; (**f**) triple-scale micro/nano structures on Cu surface [74].

Laser-induced periodic surface structures (LIPSS) are common submicron-scale self-organized structures formed on material surfaces by femtosecond laser irradiation. By adjusting femtosecond laser processing parameters, a wide range of LIPSS surface wettability features can be generated. Zemaitis et al. [68] used a femtosecond laser to fabricate LIPSS for controlling the wettability of stainless steel. When the laser energy density ranged from 0.08 to 0.64 J/cm^2^, with a scan speed of 1000 mm/s and a scan spacing of 5 μm, the contact angle of the water droplet significantly increased. The periodicity of the surface LIPSS varied when changing the laser processing parameters, causing the surface wettability to shift from superhydrophilic (with an initial water droplet contact angle of around 4°) to superhydrophobic. With further optimization of the processing parameters, the contact angle ultimately reached about 150°, successfully achieving the transition from superhydrophilic to superhydrophobic (Figure 4a). Raffaele et al. [69] studied the generation of LIPSS on quartz and their impact on the material’s wettability. By varying parameters such as laser energy density, pulse count, and repetition frequency, two types of structures, low spatial frequency LIPSS (LSFL) and high spatial frequency LIPSS (HSFL), were observed. The study found that the HSFL-textured quartz exhibited superhydrophilic behavior, with a contact angle of 7.6°. This research opens up potential applications for quartz crystals in humidity sensing. The groove structure is a typical anisotropic micrometer-scale structure. Liu et al. [53] fabricated grooves of varying widths on the surface of aluminum alloy by adjusting the femtosecond laser scanning pitch and then treated the surface with silane. As the scanning pitch increased, the groove size grew from 47 μm to 250 μm, while the ridge size remained between 14 μm and 18 μm (Figure 4b). The contact angles of all samples were greater than 130°, and as the pitch widened, the contact angles parallel and perpendicular to the groove direction gradually increased, with the difference between them rising from 2° to 10°. The anisotropic wettability is due to the water droplet being squeezed and fixed in the perpendicular direction while being stretched in the parallel direction, leading to different energy barriers in the two directions and resulting in significant wettability differences.

#### 4.1.2. Cross-Scale Structures

The microstructure and chemistry obviously affect the surface wettability. According to the Wenzel and Cassie–Baxter models, the apparent contact angle can be modified by micro/nanostructure influence when increasing the contact area or trapping air cushions. For hydrophobic surfaces with roughness factors (r) larger than 1, the contact angle can exceed 150° (Cassie state). The hydrophilic surfaces exhibit enhanced wettability due to the capillary force. The contact angle hysteresis is reduced to below 5° by cross-scale structures when trapping multiple air layers. Additionally, columnar or porous structures affect the dynamic wettability via controlling droplet spreading under the three-phase line pinning effect. Moreover, the anisotropic grooves introduce direction-dependent wettability. Developing a cross-scale structural wettability model is useful to guide the design of functional wettability surfaces for applications in anti-icing, microfluidics, and other fields.

Femtosecond laser micro/nano structuring technology enables cross-scale modification of materials. For example, micro/nano hierarchical structures constructed by femtosecond laser are composite structures consisting of both micron and nanometer scale components. The functional structures significantly reduce the contact between the droplet and the surfaces, thereby decreasing the adhesion force between the droplet and the solid surface, achieving a lower rolling angle (<10°) and a higher liquid contact angle (>150°) [70,71]. Chen et al. [72] used a femtosecond laser micro/nano processing to create micro/nano composite structures with different types of air pockets (sealed and open) on Ni surfaces. After low surface energy treatment, the surfaces exhibited superhydrophobicity, with contact angles over 150° and rolling angles below 10°, and droplets in the Cassie state. This was due to the composite structure and surface roughness, with air pockets trapping air and allowing droplets to almost hover. Anti-icing tests confirmed that the micro/nano composite structures and air pockets were key to superhydrophobicity and anti-icing performance (Figure 4c). Lin et al. [55] used a femtosecond laser to create a periodic micrometer-scale pit array on quartz glass, forming a rich nanoparticle composite structure within the pits. By adjusting pulse frequency and energy, the pit centers became porous, promoting the dispersion of more nanoparticles and the growth of numerous nanorods, significantly increasing the surface roughness and enhancing superhydrophobicity. The glass samples exhibited a contact angle of 161.2° ± 0.4° and a rolling angle of 2° ± 1°. The combination of periodic pit array and self-organized complex nanostructures enhanced surface roughness, forming and maintaining an air layer between the droplets and the glass surface, greatly improving superhydrophobic performance (Figure 4d).

#### 4.1.3. Triple-Scale Structures

In low-temperature environments, conventional micro/nano hierarchical structures are prone to failure of the Cassie wetting state, reducing the anti-icing performance of superhydrophobic surfaces and leading to ice accumulation. To address this issue, researchers have developed triple-scale micro/nanostructures, which enhance both the mechanical stability of the surface and the stability of the superhydrophobic wetting state, resulting in excellent anti-icing and de-icing performance. Xuan et al. [73] used a femtosecond laser processing combined with the boiling water treatment method to create a micro/nano hierarchical structure on an aluminum alloy substrate. This surface features periodic micro-pit arrays, non-uniform microclusters, and irregular nanosheets. By optimizing parameters such as micro-pit diameter and depth, the surface exhibits excellent superhydrophobicity, with a contact angle exceeding 150° at 0 °C and an ice adhesion strength as low as 1.60 kPa. Additionally, the surface demonstrates good anti-icing performance and mechanical durability. After 100 tape peelings, the contact angle remains above 150°, and after 48 wear cycles, the contact angle stays above 150°. After 15 ice formation and de-icing cycles, the ice adhesion strength remains below 6 kPa. This research offers a promising solution for the anti-icing/de-icing of aerospace engine blades (Figure 4e). Pan et al. [74] used a femtosecond laser to fabricate a three-layer micro/nano composite structure with a contact angle of 161.2° and a rolling angle of 0.5°, exhibiting superhydrophobic properties. The structure consists of a periodic microcone array covered with dense nanograss and scattered microflowers. The microcone array serves as the structural base, providing support for the upper nanostructures. Dense nanograss, grown in situ on the surface of the microcones, has a length between 300 and 500 nm and an average width of about 50 nm. The nanograss greatly increases surface roughness, enhancing superhydrophobicity and mechanical stability. The microcone surface and the adjacent base are dispersed with microflowers of approximately 5 μm in diameter, which further enhance the surface’s microstructural complexity and hydrophobicity. The synergistic effect of the microflowers, nanograss, and microcone structures contributes to the excellent superhydrophobic performance of the surface (Figure 4f).

### 4.2. Non-Uniform Micro/Nano Structures

**Figure 5:**
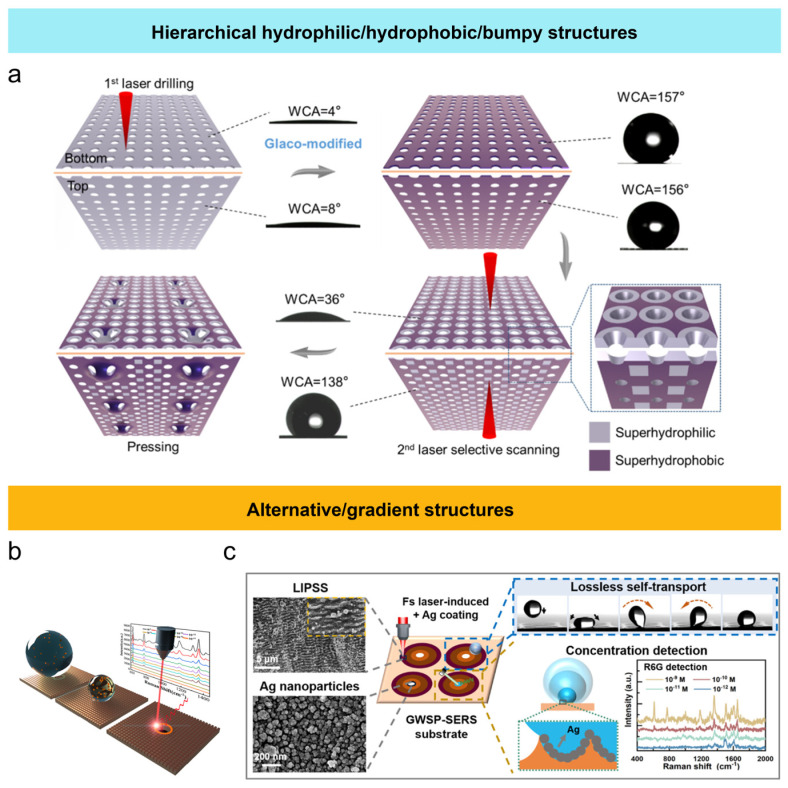
Femtosecond laser fabrication of non-uniform structures and the surface wettability: (**a**) hierarchical hydrophilic/hydrophobic/bumpy Janus membranes [77]; (**b**) alternative hydrophilic/hydrophobic structures for detecting the particle aggregation [78]; (**c**) gradient structures for droplet positioning in Raman detection [79].

In addition to uniform surfaces with single hydrophilic or hydrophobic properties, non-uniform structured surfaces combine different wettability characteristics to achieve heterogeneous wettability. Compared to uniform structures, these heterogeneous surfaces with non-uniform structures offer a wider range of functional applications, representing an extension of uniform wettability structures, opening new areas for the application of wettability surfaces [75,76]. Janus wettability membranes exhibit asymmetric surface wettability, which significantly influences fluid wetting behavior on the surface. This asymmetry enables unique fluid behaviors, such as controlling the directional movement of droplets, droplet distribution, and directed liquid transport, thus expanding their potential applications in liquid manipulation, separation, filtration, and more. Inspired by desert beetle backs and cactus spines, Su et al. [77] studied the creation of superhydrophilic surfaces on aluminum foil using femtosecond laser processing. Through chemical modification and selective laser treatment, they achieved hydrophilic–hydrophobic alternating structures to produce a hierarchical hydrophilic/hydrophobic/bumpy Janus membrane, optimized for efficient droplet transport. For example, femtosecond laser processing was applied to aluminum foil to achieve water contact angles of approximately 4° (bottom) and 8° (top). Then, chemical modification was used to transform both sides of the surface into a superhydrophobic state, with water contact angles reaching around 157° and 156°. Next, selective femtosecond laser scanning removed part of the superhydrophobic coating, exposing the hydrophilic substrate to create a hydrophilic–hydrophobic alternating structure. The membrane’s performance was optimized via the structure, enabling efficient droplet transport from the superhydrophobic top surface to the superhydrophilic bottom surface through the combined effects of surface wettability differences, chemical wetting driving forces, and the Laplace pressure difference in conical micropores. Compared to traditional Janus membranes, the hierarchical hydrophilic/hydrophobic/bumpy Janus membrane exhibited a higher fog collection efficiency in different fog flow directions, with a water collection efficiency improvement of over 250%, providing a novel approach to addressing freshwater shortages (Figure 5a). Wettability gradient and hydrophilic–hydrophobic alternating surfaces have found widespread use in SERS (Surface Enhanced Raman Spectroscopy) due to their ability to address the limitations of traditional SERS techniques in droplet positioning and enrichment. Yu et al. [78] employed femtosecond laser to first create a superhydrophobic region on the copper foil surface with a contact angle of up to 158°. Subsequently, they used secondary laser scanning to form a ring-shaped superhydrophilic (contact angle close to 0°) area within the superhydrophobic region, with micropores created at the center. The unique surface morphology formed by the hydrophilic–hydrophobic alternating structure resulted in specific behaviors of droplets during evaporation. The high adhesion force in the superhydrophilic region attracted and fixed droplets, promoting the accumulation of polystyrene nanoparticles at the superhydrophilic pattern edges, with the micropores further enhancing this enrichment effect. This design not only improved Raman testing speed but also advanced the practical application of SERS in ultra-trace molecular detection (Figure 5b). Li et al. [79] controlled femtosecond laser parameters to create LIPSS on a copper substrate, achieving a wettability gradient structure. The surface gradually transitioned from superhydrophobic to hydrophilic from the outer to the inner region, with the outermost contact angle reaching 158° and the central region having a contact angle of less than 10°. This wettability gradient allowed the test droplets, regardless of their initial position, to quickly and accurately self-transport to the central detection area under the Laplace pressure generated by the surface tension difference. This design effectively solved the droplet positioning challenge, reduced solution waste, and enhanced the accuracy and reliability of detection, improving SERS detection efficiency (Figure 5c).

## 5. Femtosecond Laser Modulation for Multi-Scale Structure Fabrication

Femtosecond laser processing, with its ability to create complex patterns, combines the characteristics of femtosecond laser ablation and the inherent properties of different materials. By adjusting processing parameters such as energy density, scanning speed, frequency, scan count, and scanning spacing, it is possible to fabricate micro/nano structures with various morphological features, opening new avenues for creating complex and customized surface wettability properties. While the laser beam spatially propagates, three physical parameters usually serve to describe the energy distribution, mainly including the intensity, phase, and polarization. Therefore, the high-precision cross-scale micro/nano structures can be formed by adjusting the appropriate spatial laser energy, thereby breaking down the trade-off between the quality and efficiency.

### 5.1. Spatial Light Modulation

Spatial light modulation (SLM) is attracting more attention to adjust one or more physical parameters in the two-dimensional light field, relying on the digital micromirror devices (DMDs), liquid crystal spatial light modulators (LC-SLM), or meta-surfaces. Using SLM, the shape and polarization can be accurately modified for more complex structures and improved processing efficiency. Controlled by the adjusting voltage, the main modulation for femtosecond laser mainly consists of shape, frequency, pulser, polarization modulation, and so on.

**Figure 6:**
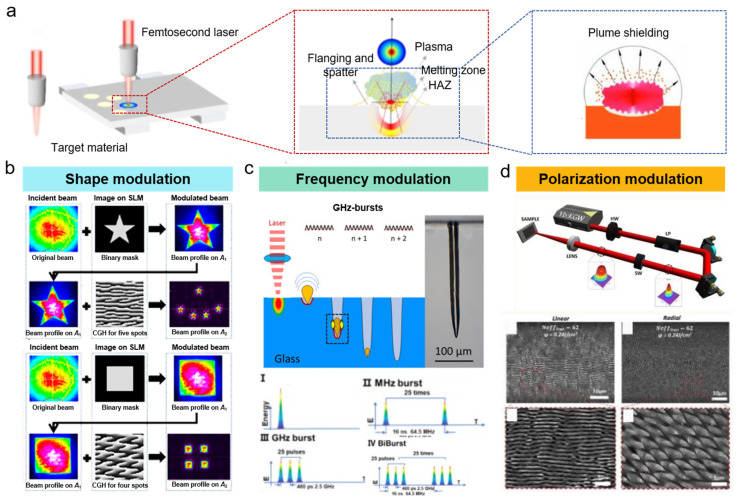
(**a**) Plume shielding during femtosecond laser fabrication [85]; (**b**) shape modulation for processing of complex structure [80]; (**c**) frequency modulation for MHz/GHz burst mode [86,87]; (**d**) polarization modulation and processed structures with linear and radial polarization [90].

*Shape Modulation*: The widespread shape modulation for femtosecond laser mainly transforms from Gaussian laser to flat-top laser, slit laser, vortex laser, and Bessel laser. As seen in Figure 6b, the beam shaping can be successfully transferred to the high-quality desired beam and realized based on the plano-convex cylindrical mirror [80], assisting the fabrication of high aspect ratio structures, such as deep drilling, dielectric cleaving, cutting, and so on. Moreover, Cheng et al. [81] introduced a digital micromirror device (DMD)-based ultrafast beam shaper and generated the arbitrary beam with 1140 × 912 pixels at the repetition rate of 4.2 kHz. The laser shape has demonstrated playing a great role in the femtosecond laser ablation process, considering the fabricating characteristics and processing efficiency. For example, Zhang et al. [82] achieved width-controllable surface grooves with uniform bottom and side wall steepness using the flat-top femtosecond laser beam. Moreover, the higher-order Bessel or Bessel-like laser beam is superior in the laser drilling of high-aspect-ratio holes for improving the hole taper, largely ascribed to the tunable and longer non-diffraction length. By using the optimized Bessel pulse energy, the aspect ratio of microholes exceeded 460 [83]. Tan et al. [84] also demonstrated that the slit shaping achieved by adjustable mechanical slits was positive for the femtosecond laser fabrication of 2D waveguide arrays with depth-insensitive, circular cross-sections and low loss. The lateral and longitudinal aspect ratios were all increased from an initial 0.2 to about 1.

*Frequency Modulation*: Repetition frequency serves as a crucial role in the femtosecond laser ablation. Compared with traditional low frequency, the femtosecond laser with higher frequency is proved to enhance the ablation depth and efficiency by increasing the number of pulses, ensuring relatively larger-scale material removal. On the other side, the “plume shielding” (seen in Figure 6a) may hinder the energy absorption of the subsequent pulse and weaken the ablation precision for excessively high repetition frequency [85]. Especially, the accumulation of multi-pulse for femtosecond laser can significantly lift the ablation efficiency, accompanied by more plasma, energetic shockwaves, and liquid expulsion. By temporal modulation, the burst mode can be achieved for a MHz/GHz femtosecond laser when employing the burst regime, and the number of pulses per burst is positively linear to the material removal rate when the individual pulses within the burst do not exceed the material ablation threshold (Figure 6c). For example, Balage et al. [86] achieved top-down percussion drilling of blind holes and avoided an obvious ejected plume when employing the burst repetition rate of 1 kHz for a femtosecond laser with a frequency of 1.28 GHz, largely attributed to the heat accumulation induced by the high pulse repetition rate and the elevated pulse number. The ablation region was mainly dependent on the interaction between the plumes composed of hot, dense matter. Meanwhile, Balage et al. [87] also utilized a Bessel femtosecond laser for directly cutting glasses up to 1 mm in thickness and achieved submicron-scale surface roughness Sa of the cutting planes in the GHz-burst mode.

*Polarization Modulation*: The time-dependent polarization of a femtosecond laser pulse usually depends on a pulse shaper and contours the problems, such as precisely controlling the propagation of light at short wavelengths. Tunable polarization can be achieved when using the externally seeded free-electron laser (FEL) FERMI, generating two mutually delayed, phase-locked, cross-polarized FEL sub-pulses (counter-rotating circular polarizations or perpendicular linear polarizations) by introducing polarization-dependent changes in optical phase. Kuo et al. [88] investigated the role of polarization separation on the femtosecond laser grooving and demonstrated that the greater depth and smoother sidewalls could be achieved by the radially polarized beam, and the polarization had a close relationship with the processing direction. Li et al. also [89] proposed that the high purity (94.7%) longitudinal femtosecond broke down the optical diffraction limits and prepared holes of 10–30 nm in diameter with a depth-to-width aspect ratio of over 16 and zero taper. Furthermore, Zhang et al. [90] employed circularly polarized or linear cross-polarized femtosecond laser to fabricate the supra-wavelength periodic surface structures (SWPSS) and found that the orientation of SWPSS patches was completely independent of the light polarization direction (Figure 6d).

### 5.2. Modeling for the Modulated Femtosecond Laser Processing

Due to the complex physical phenomenon during modulated femtosecond laser processing of various structures on materials, it is essential to understand the dynamic process between the modulated and the matter based on multi-scale and multi-physics coupled modeling to investigate the spatial energy distribution and further morphology evolution.

**Figure 7:**
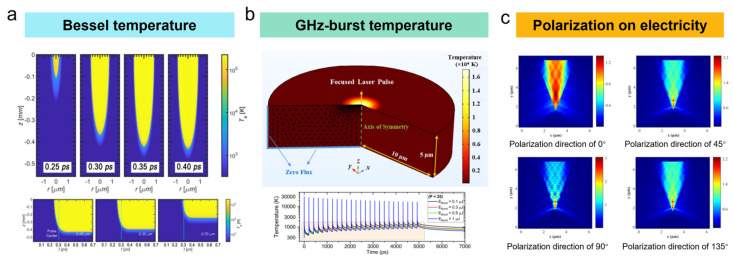
(**a**) Temperature profile during Bessel femtosecond laser processing [91]; (**b**) temperature profile during femtosecond laser processing at GHz-burst mode [92]; (**c**) electronic field distribution at different polarization directions [96].

For the shape modulation, a significant boundary formed at the edge of the focused region is usually introduced to address the energy distribution issue for the modulated laser. For example, Gong et al. [91] numerically discussed the interaction between Bessel beam and α-quartz, especially the absorption and reflection of laser energy (Figure 7a). The optical penetration depth at the edge of the Bessel beam irradiation region rapidly drops to hundreds of nanometers. The low laser intensity in the center and high laser intensity on the edge occur. For the frequency modulation, Momeni et al. [92] developed a two-dimensional axisymmetric model considering the GHz burst mode and captured the transient temperature profile and crater formation during femtosecond laser irradiation (Figure 7b). The simulated results quantitatively agreed with the experimentally measured ablation depth and demonstrated that higher efficiency in ablation was obtained by the GHz burst mode. The irradiated material is locally melted and removed rather than evaporated under GHz femtosecond laser ablation. Cheng et al. [93] also established an axisymmetric model to investigate the temperature field under the interaction of GHz femtosecond laser with aluminum film by adopting classical TTM (Two Temperature Models, TTM) considering the latent heat of phase change from solid to liquid phase. The surface morphology shows the transition from melting-dominated course to mixed melting and evaporation. For the polarization modulation, Su et al. [94] proposed that the degree of polarization had an effect on the ultrashort laser transmission and the pulse compression, and the linear, elliptic, and circular polarizations showed various intensity distributions due to the self-focusing effect. Skolski et al. [95] demonstrated the polarization of the material surface after laser irradiation using the Sipe model and the FDTD model. Wang et al. [96] analyzed the polarization on the electric field intensity distribution within the groove based on the finite-difference-time-domain (FDTD) method. The electric field strength distribution is entirely different, and the laser energy is concentrated inside the groove (Figure 7c), leading to a larger groove taper at the polarization angle of 0°.

## 6. Application of Wettability Control in Industry Fields

### 6.1. Environmental Engineering

**Figure 8:**
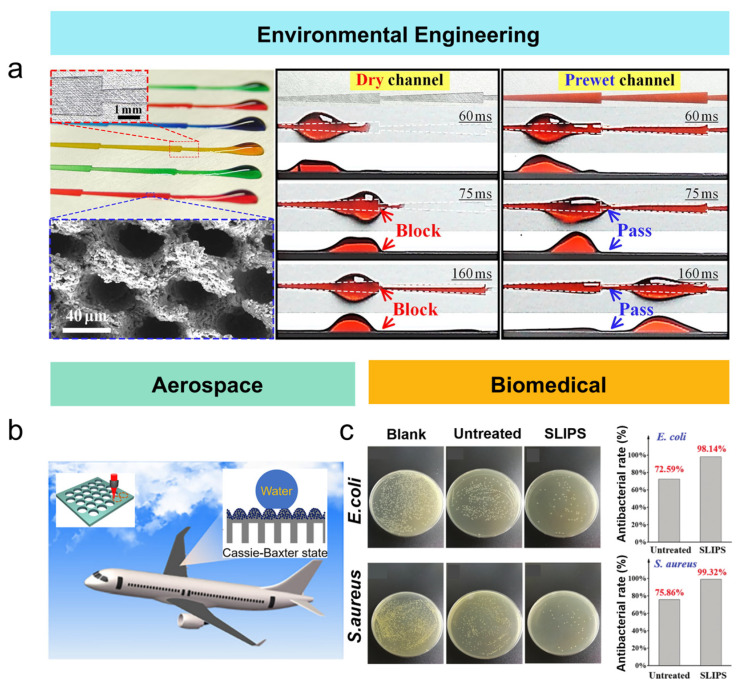
Applications of femtosecond laser-treated wettability-functional materials in various fields: (**a**) environmental engineering [97]; (**b**) aerospace [100]; (**c**) biomedical fields [103].

Superhydrophobic surfaces with high viscosity fabricated by femtosecond laser can transport droplets without damage. Droplets are transferred from low-viscosity to high-viscosity surfaces with minimal liquid loss, offering great potential for industrial wastewater treatment. Liu et al. [97] used a femtosecond laser scanning on aluminum plates and obtained superhydrophobic surfaces after secondary laser etching. The patterns provide wedge-shaped channels, allowing water droplets to be quickly transported in a specified direction under Laplace pressure. Meanwhile, oil spills and oily wastewater may result in significant pollution and can be addressed by efficient oil–water separation. By leveraging wettability differences, femtosecond laser can achieve oil–water separation by surface structuring (Figure 8a). Yong et al. created perforated PTFE films with superhydrophobic surfaces using mechanical processing combined with femtosecond laser ablation [98]. The superoleophilic surface allowed oil to pass through while the superhydrophobic surface trapped water, enabling successful oil–water separation. On the other hand, collecting methane bubbles that escape from the seabed or lake water can help alleviate the energy crisis. Underwater superhydrophobic/oleophilic porous membranes can selectively trap bubbles, achieving efficient water-gas separation. Yao et al. [99] employed femtosecond laser to create 54 μm micropores on PTFE tubes and achieved the superhydrophobic and underwater superoleophilic via micro/nano rough structures. When underwater bubbles contact the tubes, Laplace pressure drives the gas in the bubbles through the micropores into the tube, allowing for efficient water-gas separation.

### 6.2. Aerospace

In the aerospace field, the risk of icing in low-temperature environments acts as the bottleneck when flying at high altitudes. Inspired by the thermally insulating hollow structures of polar bear fur and the anti-reflective micro/nano structures of mosquito compound eyes, Xuan et al. [100] combined femtosecond laser ablation with template transfer technology to fabricate a micro/nano hollow film. By optimizing the femtosecond laser scanning distance, the micro/nano structures were successfully adjusted, achieving a surface contact angle of approximately 163.8° and a rolling angle of about 0.5°, demonstrating superhydrophobicity. The bionic composite structures exhibit superhydrophobicity and introduce dual-layer optical traps to enhance light utilization efficiency. The internal hollow micro-column structures effectively promote light absorption and block heat conduction, significantly reducing the reflection and transmission intensity. Furthermore, the material demonstrates excellent flexibility, mechanical durability, chemical stability, and self-cleaning capability. After 10 freeze–thaw cycles, the material maintains superhydrophobicity with a low ice adhesion strength of 1.8 ± 0.3 kPa, showcasing the immense potential for applications in aircraft wing de-icing (Figure 8b). The influence mechanism of contact angle on the anti-icing performance of superhydrophobic surfaces can be summarized in three key aspects: First, a larger contact angle increases the energy barrier for ice nucleation and reduces the solid–liquid contact area, thereby significantly delaying ice formation. Second, the low contact angle hysteresis of superhydrophobic surfaces promotes the rapid detachment of supercooled water droplets before ice nucleation. The air film trapped in the micro/nanostructures reduces solid–liquid heat transfer efficiency, further accelerating droplet rebound. Finally, the synergy of high contact angle and low adhesion reduces the ice–substrate interfacial bonding strength.

### 6.3. Biomedical

When the metal implants contact blood, coagulation and thrombosis are the inevitable issues. By femtosecond laser precisely controlling the micro/nano structures on the surface, the anti-coagulation and anti-thrombosis properties can be significantly improved, thereby enhancing the safety and performance of metal implants in biomedical applications. For example, He et al. [101] used a femtosecond laser to process micro-groove and micro-protrusion structures on titanium surfaces, investigating the surface morphology, wettability, and biocompatibility. The results showed that femtosecond laser-induced microstructures obviously promoted cell adhesion. Xiao et al. [102] fabricated ordered microstructures based on femtosecond laser on Ti6Al4V alloy. By optimized laser energy density of 2.31 J/cm^2^ and scanning pass of 50, regular microstructures were formed on the titanium surface, significantly enhancing the hydrophilicity. The application of femtosecond laser surface modification on metal medical implants effectively improves biocompatibility, offering a novel pathway for the medical field. Cheng et al. [103] proposed a method to fabricate SLIPS on NiTi alloys to improve their hemocompatibility. As shown in Figure 8c, after culturing Escherichia coli and Staphylococcus aureus, the number of bacteria on the SLIPS is much less than that on the untreated NiTi alloy. The antibacterial rates reach 98.14% and 99.32%, respectively, significantly enhancing the antibacterial performance. In addition, the SLIPS has good anticoagulation properties and a low hemolysis rate, greatly improving the hemocompatibility of the alloy. This research provides a new approach to improving the safety and effectiveness of NiTi alloys in medical applications and is expected to promote the development of metal implant materials (Figure 8c).

In summary, the femtosecond laser-based wettability control has been demonstrated to be positive for the biocompatible, aerospace, and environmental engineering fields, offering high precision and material compatibility. However, the further industrialization still faces several challenges, such as high equipment costs and low processing efficiency. From the technical perspective, inadequate control of laser energy distribution in both time and space leads to reduced structural consistency over a large area. Moreover, the processing stability for large-scale complex micro/nanostructures is challenging. The multi-beam parallel processing and adaptive light field control provide a novel insight into improving efficiency and scalability, enabling broader industrial applications.

## 7. Summary and Outlook

This paper summarizes the recent research progress referenced to wettability micro/nano structures for surface wettability control and the related applications based on the femtosecond laser ablation. Known for the high precision, non-contact operation, feasible controllability, and broad material adaptability, femtosecond laser processing has emerged as an effective approach for fabricating wettability-functional micro/nano structures and has been widely employed in the environmental engineering, aerospace, and biomedical fields. For hydrophilic materials with high surface energy, femtosecond laser processing can create micro/nano structures to enhance superhydrophilicity. However, these surfaces are susceptible to organic adsorption, leading to a transition to hydrophobicity. To address this, low-surface-energy coatings can be applied to improve stability. For low-surface-energy hydrophobic materials, femtosecond lasers can further enhance superhydrophobicity. Additionally, smart wettability-switching materials have enabled reversible wettability modulation through external fields (such as heating, pressure, or light), providing new application prospects for multifunctional surfaces. Uniform micro/nano structures, such as single and hierarchical structures, allow precise control on the surface wettability, such as ranging from superhydrophobicity to superhydrophilicity by adjusting femtosecond laser parameters. Non-uniform structures, including Janus membranes and gradient surfaces, show superiority in droplet transport, separation, and surface-enhanced Raman spectroscopy (SERS). Based on the shape, frequency, and polarization modulation, femtosecond laser shows superiority in the quality and efficiency of the multi-scale structure fabrication, even breaking down the optical diffraction limits. Future research on femtosecond laser-treated wettability-functional surfaces should focus on two key aspects. First, in terms of cross-scale structures, combining techniques like self-assembly, femtosecond laser processing, and chemical etching can create high-precision nanoscale structures (<100 nm) and mesoscopic functional units to precisely control droplet transport. Second, attention should be given to the service performance of these surfaces, particularly by revealing surface failure mechanisms under mechanical and chemical loading and developing stable superhydrophobic/hydrophilic surfaces with in situ repair capabilities. Moreover, advanced mesoscopic designs and large-scale formation of complex surfaces will broaden the technology’s scope and improve engineering feasibility.

## Figures and Tables

**Table 1:** Summary of materials for femtosecond laser processing of wettability-functional surfaces.

Target		Frequency (kHz)	Wavelength (nm)	Pulse Width (fs)	Laser Energy	WCA (°)	Ref.
Intrinsic hydrophilic materials	Ni	1	800	50	1–2 mJ	153.0	[52]
	Al	400	1030	400	−	>150.0	[53]
	Stainless steel	0.5	800	35	1.6 mJ	156.9	[54]
	Silica glass	200	1030	800	100 μJ	161.0	[55]
	Al	75	1030	1000	1–10 W	163.5	[56]
	Cu	200	1030	800	7.08 J/cm^2^	>150.0	[57]
Intrinsic hydrophobic materials	PTFE	1	800	140	51 J/cm^2^	170.0	[58]
	PTFE	75	1030	250	11 W	157.0	[59]
	PTFE	1	800	300	25 mW	158.6	[60]
	PDMS	1	800	50	30 mW	156.0	[61]
	PDMS	1	800	100	0.72 J/cm^2^	155.4	[62]
	PDMS	1	800	104	350 mW	174.0	[63]
	Rubber	1	800	100	136.2 J/cm^2^	153.1	[64]
	PEEK	100	1035	350	900 mW	165.2	[65]
Smart materials with switching wettability		1	800	50	30 mW	153.5	[49]
		1	800	50	100 mW	156.3	[66]
		1	800	50	30 mW	152.8	[67]
